# Automatic versus manual changeovers of norepinephrine infusion pumps in critically ill adults: a prospective controlled study

**DOI:** 10.1186/s13613-015-0083-7

**Published:** 2015-11-14

**Authors:** Emilie Greau, Jean-Baptiste Lascarrou, Aurélie Le Thuaut, Nathalie Maquigneau, Yolaine Alcourt, Anne Coutolleau, Cécile Rousseau, Vanessa Erragne, Jean Reignier

**Affiliations:** Medical-Surgical Intensive Care Unit, District Hospital Center, La Roche-sur-Yon, France; Clinical Research Unit, District Hospital Center, La Roche-sur-Yon, France; Délégation à la Recherche Clinique et à l’Innovation, CHU Hôtel Dieu, 44093 Nantes Cedex, France; UPRES EA-3826, Clinical and Experimental Therapies for Infections, University of Nantes, Nantes, France; Medical Intensive Care Unit, Nantes University Hospital, Nantes, France

**Keywords:** Critically ill, Shock, Changeover, Catecholamines, Norepinephrine, Smart infusion pumps

## Abstract

**Background:**

Norepinephrine is a key drug for treating shock but has a short half-life that requires continuous intravenous administration to maintain the constant plasma concentration needed to obtain a stable blood pressure. The small volume of the syringes used in power infusion pumps requires frequent changeovers, which can lead to norepinephrine flow interruptions responsible for hemodynamic instability. Changeovers from the nearly empty to the full syringe can be performed manually using the quick change technique (QC) or automatically using smart infusion pumps (SIP) that link two syringes. The purpose of our study was to evaluate the hypothesis that, compared to QC, SIP for norepinephrine changeovers was associated with less hemodynamic instability.

**Methods:**

After information of the patient or next of kin, patients receiving norepinephrine for shock were allocated to QC or SIP changeovers. QC changeovers were performed by a nurse, who started a new loaded pump when the previous syringe was nearly empty. SIP changeovers were managed automatically by SIP workstations. The primary outcome was the proportion of changeovers followed by a ≥20 % drop in mean arterial pressure (MAP).

**Results:**

411 changeovers were performed, 193 in the 18 patients allocated to QC and 218 in the 32 patients allocated to SIP. Baseline patient characteristics were similar in both groups. The proportion of changeovers followed by an MAP drop ≥20 % was 12.4 % (24/193) with QC and 5.5 % (12/218) with SIP (*P* = 0.01). By multivariate analysis, two factors were independently associated with a significantly decreased risk of ≥20 % MAP drops during changeovers, namely, SIP (odds ratio, 0.47; 95 % confidence interval, 0.22–0.98) and norepinephrine dosage >0.5 μg/kg/min (odds ratio, 0.39; 95 % confidence interval, 0.19–0.81).

**Conclusions:**

The risk of MAP drops ≥20 % during changeovers can be significantly diminished using SIPs instead of the QC method.

Trial registration: Clinicaltrial.gov NCT 01127152

## Background

The management of hemodynamic failure, a key issue in intensive care, requires close and continuous monitoring and treatment. Vasoactive drugs are used to reverse vasoplegia and/or to treat heart failure in patients with persistent hemodynamic instability after volume repletion to correct hypovolemia [1–3]. Norepinephrine is currently the most widely used drug to treat vasoplegia, because of its potent and selective effects on vascular alpha receptors [4]. Similar to all other vasoconstrictors, norepinephrine has a short half-life. These features require intravenous administration at a constant flow rate, which can be achieved using a high-precision power infusion pump [5, 6].

The syringes used with power infusion pumps are small and must therefore be changed frequently. The changeover between vasoactive drug infusion pumps (CVIP) can induce a brief change in the drug flow rate manifesting as hemodynamic instability [7]. Various methods for performing manual or automatic changeovers have been developed to prevent episodes of hemodynamic instability [6, 8]. In the quick change method (QC), a full syringe is loaded into a new pump then connected to the three-way stopcock, which is turned to open the new and close the old infusion. This new primed line is set at the same flow rate as the running pump. QC has been proven simple and effective for manual CVIP [7, 9, 10] but does not completely eliminate the risk of instability. In addition, QC generates considerable work and stress for the nurse.

Smart infusion pumps (SIP) linking two syringes have been developed to allow automatic CVIP without changing the flow rate [11]. The SIP software has a number of capabilities, including a relay feature that can link a full syringe and a nearly empty syringe, thereby limiting flow rate interruptions during CVIP while decreasing the need for nurse interventions. Compared to manual CVIP, automated CVIP using an SIP may therefore minimize episodes of hemodynamic instability. A before–after study showed reduced rates of hemodynamic incidents and decreased nurse workload with SIP compared to QC [12]. However, the available data are scant, and no randomized trials or guidelines are available.

The purpose of this prospective controlled trial was to investigate whether using a SIP for norepinephrine changeovers decreased hemodynamic instability compared to QC.

## Methods

### Study design and setting

This prospective controlled trial was conducted from June 2009 to February 2011 in the 18-bed adult medical-surgical intensive care unit (ICU) of the District Hospital Centre in La Roche-sur-Yon, France. The study protocol was approved by the appropriate ethics committee (Comité de Protection des Personnes de Poitiers) on April 15, 2009. According to French law, because the strategies used in both study groups are considered standard care, there was no requirement for signed informed consent. However, information of the patient or next of kin was required before inclusion in the study. The patient or next of kin could decline participation in the study at any time after being informed or included.

### Patients

Adults (age > 18 years) admitted to the ICU and given norepinephrine to treat shock were eligible for inclusion. Exclusion criteria were moribund status, pregnancy or breastfeeding, infusion of another medication via the central venous port used for norepinephrine administration, absence of healthcare insurance, refusal of the patient or next of kin to participate in the study, pheochromocytoma, and carcinoid tumor.

### Allocation

Eligible patients were informed about the study then enrolled by study nurses, who randomly allocated them in a 1:1 ratio to the QC or SIP method for CVIP, as described below. The random allocation sequence was generated by the clinical research unit of the La Roche Sur Yon District Hospital, using Random© (Inserm, Paris, France). No stratification was performed. Implementation of the sequence was by opaque sealed envelopes to ensure concealment.

### Norepinephrine administration

**Fig. 1:**
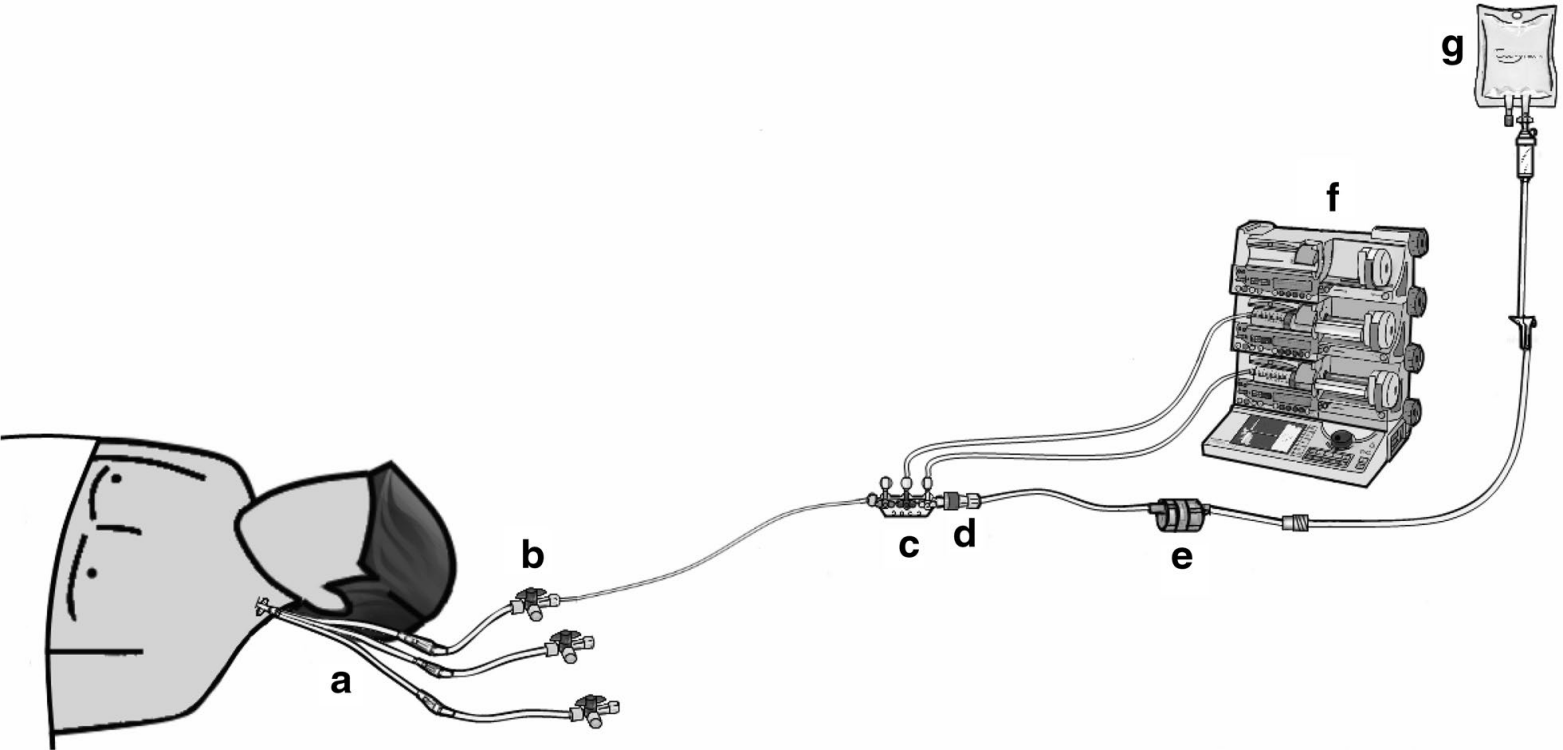
Infusion line setup. **a** Multilumen central venous catheter (Arrow, Kingston, UK); **b** infusion line (BD Medical, Le Pont de Clais, France) connected to the proximal lumen of the central venous catheter; **c** perfusion ramp with a three-way stopcock (Vygon, Ecouen, France); **d** anti-reflux valve (Arrow, Kington, UK); **e** infusion rate control (CAIR LGL, Lissieu, France); **f** infusion pump; **g** Dextrose 5 % infusion set (B. Braun, Boulogne Billancourt, France)

Infusion lines and norepinephrine syringes were prepared according to the written protocol of the unit. The infusion lines were similar in both groups (Fig. 1). All devices used for the study were those used habitually in the unit. All ICU nurses were trained in using power infusion pumps, setting up infusion lines, and preparing norepinephrine syringes. They attended a training program specifically designed to ensure optimal quality and standardization of QC and SIP changeovers. These training programs were delivered by three highly experienced ICU nurses, who were in charge of bedside nurse training in our unit. Written standardized protocols for QC and SIP changeovers were developed, and the ability of each ICU nurse to follow them successfully was checked by the training nurses.

All study patients received continuous norepinephrine via a dedicated infusion line (BD Medical, Le Pont de Clais, France) connected to the proximal lumen of a multilumen central venous catheter (Arrow, Kingston, UK). Each Luer-Lock 60-mL syringe was loaded with an amount of norepinephrine, in mg, equal to the patient’s body weight multiplied by 0.3; water for intravenous injection (Renaudin, Itxassou, France) was then added to obtain a total volume of 50 mL. The NE ampoules used in our unit to prepare the syringes contained 8 mg of NE. When the calculated NE requirement was between 23 and 25 mg, the nurses rounded to 24 mg (three ampoules) to increase the efficiency and speed of preparation at the bedside. The infusion rate in μg/kg/min was equal to 1/10 of the injection speed of the power infusion pump. Changeovers were performed when the syringe contained 5 mL or less; finally, as a safeguard, the electric infusion pump triggered a buzzer when less than 2 mL of the norepinephrine solution was left in the syringe. In both groups, a 5 % dextrose carrier of the norepinephrine infusion was used [13]. It was infused by gravity with a flow rate regulator (Fig. 1). The flow was set at 5 mL/h for all patients and checked regularly by the nurse at the bedside [14].

#### Changeovers in the QC group

Orchestra^®^ power pumps were used (Fresenius Kabi, Bad Homburg, Germany). The patient’s nurse prepared a new syringe with the same concentration of norepinephrine, connected it to a line and to a new power pump set at the same flow rate as the running pump, primed the line using the bolus function of the pump until a drop of norepinephrine solution was visible at the tip of the line (thereby minimizing the start-up time of the new infusion), then connected the line to the three-way stopcock. When the previous syringe contained 5 mL or less of norepinephrine solution, the nurse turned the stopcock to stop the old infusion and start the new infusion. Throughout this procedure, the patient was monitored closely by the nurse, who remained at the bedside.

#### Changeovers in the SIP group

The Orchestra^®^ Base Intensive SIP workstation (Fresenius Kabi) was used. This device can manage up to six power infusion pumps and can perform automated changeovers by driving one syringe after the other at the same flow rate. The nurse presses the changeover button on the workstation, causing the display to divide into two windows, one for each syringe. The nurse then loads the new syringe containing norepinephrine solution in the same concentration as the running syringe. The new syringe is primed using the bolus function of the pump until a drop of norepinephrine solution appears at the end of the new line. Then, the new infusion line is connected to the three-way stopcock, which is then opened to both lines. After validation by the nurse, the workstation display shows the word “relay”, indicating that the device is ready to perform the changeover. When the relay occurs, a buzzer sounds to let the nurse know that blood pressure should be monitored.

### Data collection and follow-up

We collected the following baseline patients characteristics: age, gender, weight, body mass index, admission diagnosis, Simplified Acute Physiology Score II (SAPS II score; range from 0, lowest level of critical illness to 163, most severe level of critical illness) [15], cause of shock, Sepsis-related Organ Failure Assessment score (SOFA score; range from 0, no organ failure to 24, highest level of multiple-organ failure) [16], mechanical ventilation, ICU length of stay, and ICU and hospital mortality. For each CVIP, we collected the SOFA score, the norepinephrine dose (μg/kg/min) and concentration (mg/50 mL), blood pressure, and heart rate (HR). Noninvasive blood pressure monitoring has been proven accurate in detecting MAP drops [17], and invasive monitoring via an arterial catheter was therefore not required by the study protocol. Blood pressure was monitored continuously if the patient had an arterial catheter for another reason (Arrow, Kingston, UK) and at 1-min intervals using an automatic sphygmomanometer (Philips, DA Best, Netherlands) with an appropriately sized cuff otherwise. Maximal and minimal values of HR and MAP were recorded every 5 min over a period ranging from 15 min before to 15 min after each changeover.

### Study outcome

The primary outcome was the proportion of changeovers followed by an MAP drop ≥20 % within 15 min after the changeover. The 15 min were counted starting at the stopcock turn in the QC group and the buzzer in the SIP group. Secondary outcomes were the proportions of changeovers followed by a ≥15 % MAP drop, a ≥10 % MAP drop, severe hypotension defined as MAP <50 mmHg, and a ≥20 % HR change in either direction, within 15 min after the changeover.

### Sample size

We expected an MAP drop ≥20 % to occur within 15 min after 20 % of QC procedures [7]. To detect a 10 % decrease in the primary outcome in the SIP group, with α set at 0.05 and 80 % power, 200 changeovers were required in each group. All changeovers in each patient were included in the study. Patients on norepinephrine therapy usually require about 10 changeovers in all [7]. Therefore, about 40 patients in all (20 per group) were required. Because some patients require fewer than 10 changeovers and missing data may occur for some changeovers (e.g., those performed on an emergency basis), we planned to include 50 patients in all. The number of relays was checked during the enrollment phase of the study. After inclusion of 36 patients (18 in each group), 193 relays were available for analysis in the Quick Change Method compared to only 141 in the Smart Infusion Pump group. At this point, we decided to include all subsequent patients in the Smart Infusion Pump group until the required number of changeovers was reached.

### Statistical analysis

Quantitative data were described as means (±standard deviation) or medians (25th–75th quartiles) and compared between changeover groups using the Student or Wilcoxon–Mann–Whitney tests, as appropriate. Qualitative data were described as *n* (%) and compared using the Pearson Chi-square test or Fisher exact test as appropriate.

A mixed-effect logistic regression model was fitted to assess factors associated with the primary outcome (≥20 % MAP drop within 15 min after the changeover). This model took the hierarchical structure of the data into account. We used a random-intercept logistic model with changeovers (level 1) nested in patients (level 2). Potential confounders related to changeover characteristics were tested. Potential confounders were identified according to the results of the univariate analysis and a previous study [12]. Covariates associated with *P* values <0.10 by univariate analysis were entered into the multivariate model. Norepinephrine concentration and dose, SOFA score, and age were forced into the final multivariate model. Odds ratios (ORs) and their 95 % confidence intervals (95 % CIs) were estimated. The same method was used to analyze ≥20 % HR variations and other secondary outcomes.

All comparisons were two-sided, and *P* values <0.05 were considered significant. The data were analyzed using Stata statistical software version 13.0 (StataCorp LP, College Station, TX, USA).

## Results

**Fig. 2:**
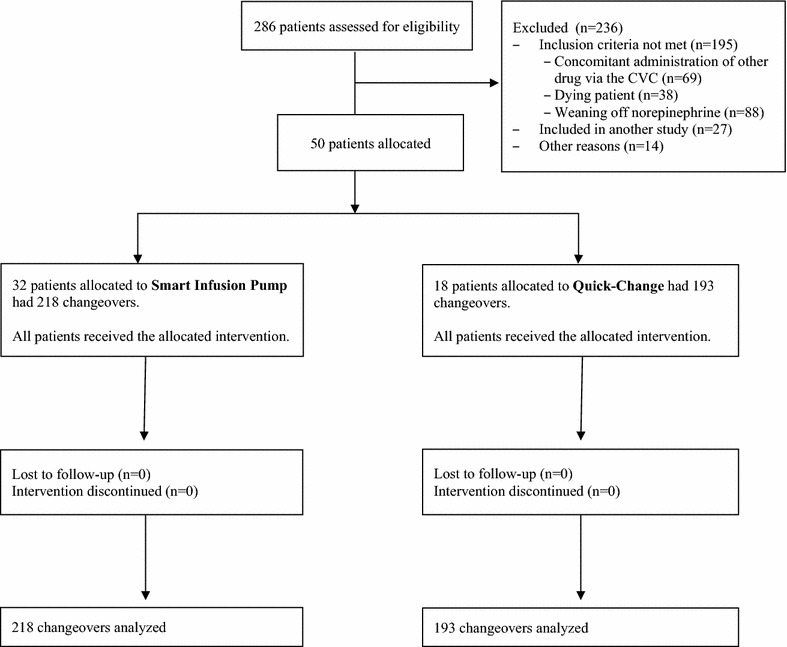
Flowchart

The 50 included patients had 411 changeovers, 193 in the 18 patients allocated to QC and 218 in the 32 patients allocated to SIP (Fig. 2). The median number of changeovers per patient was 6 [3–12] in the QC group and 3 [1–11] in the SIP group (*P* = 0.07). All patients had at least 1 changeover. The only patient characteristic that differed significantly between the two groups was ICU stay length, which was longer in the QC group (Table 1). Table 2 compares the baseline characteristics in the two changeover groups; the norepinephrine dose and HR were significantly higher in the SIP group than in the QC group.

**Table 1:** Patient characteristics

	Quick change method (*n* = 18)	Smart infusion pump (*n* = 32)	*P* value
Age (years), mean ± SD	63.3 ± 13.4	68.1 ± 12.8	0.22
Gender (male), *n* (%)	14 (77.8 %)	25 (78.1 %)	0.98
Weight (kg), mean ± SD	77.7 ± 14.1	86.5 ± 19.9	0.10
Body mass index (kg/m^2^), mean ± SD	27.7 ± 5.5	29.9 ± 5.0	0.36
Admission diagnosis, *n* (%)			0.26
Cardiac arrest	2 (11.1 %)	3 (9.4 %)	
Circulatory failure	9 (50 %)	9 (28.1 %)	
Acute respiratory failure	3 (16.7 %)	14 (43.8 %)	
Trauma	0	1 (3.1 %)	
Other	4 (22.2 %)	5 (15.6 %)	
Surgical patient, *n* (%)	8 (44.4 %)	8 (25.0 %)	0.16
SAPS II, mean ± SD	64.1 ± 19.3	65.9 ± 18.6	0.75
Method of arterial pressure monitoring			0.72
Automatic sphygmomanometer	3 (16.7)	8 (25.0)	
Invasive arterial line	15 (83.3)	24 (75.0)	
Cause of shock, *n* (%)			0.78
Sepsis	14 (77.8 %)	24 (75.0 %)	
Hemorrhage	2 (11.1 %)	2 (6.4 %)	
Other	2 (11.1 %)	5 (16.1 %)	
SOFA score at inclusion, mean ± SD	12.5 ± 4.0	11.3 ± 2.7	0.21
Mechanical ventilation, *n* (%)	17 (94.4 %)	29 (90.6 %)	0.63
ICU length of stay (days), median (IQR)	15 [9–34]	9 [3–25.5]	0.03
ICU mortality, *n* (%)	5 (27.8 %)	13 (40.6 %)	0.36
Hospital mortality, *n* (%)	5 (27.8 %)	14 (43.7 %)	0.26

*SAPS II* Simplified Acute Physiology Score, version II, *SOFA* Sequential Organ Failure Assessment, *ICU* intensive care unit, *IQR* inter-quartile range

**Table 2:** Baseline characteristics in the two changeover groups

	Quick change method (*n* = 193)	Smart infusion pump (*n* = 218)	*P* value
Age in years, mean ± SD	64.9 ± 12.2	65.7 ± 15.0	0.71
Male, *n* (%)	177 (91.7 %)	204 (93.6 %)	0.47
Weight, mean ± SD	78.8 ± 15.5	81.6 ± 18.5	0.84
Body mass index (kg/m^2^), mean ± SD	26.7 ± 5.7	28.6 ± 4.2	0.13
Cause of shock, *n* (%)			0.34
Sepsis	176 (91.2 %)	194 (90.6 %)	
Hemorrhage	10 (5.2 %)	7 (3.3 %)	
Other	7 (3.6 %)	13 (6.1 %)	
SOFA score, mean ± SD	13.3 ± 3.1	12.9 ± 3.1	0.2
Mechanical ventilation, *n* (%)	186 (96.4 %)	215 (98.6)	0.14
Dose of NE, µg/kg/min, median [IQR]	0.6 [0.3–1.2]	0.8 [0.4–2.0]	<0.001
Concentration of NE, mg/50 mL, mean ± SD	25.1 ± 6.9	24.8 ± 6.3	0.34
Heart rate at baseline	99.3 ± 22.1	107.2 ± 18.9	<0.001
Mean arterial blood pressure at baseline	78.2 ± 13.8	76.8 ± 13.4	0.31

*SOFA* Sequential Organ Failure Assessment, *NE* norepinephrine, *IQR* inter-quartile range

### Primary outcome

MAP dropped by ≥20 % in 24/193 (12.4 %) QC changeovers and 12/218 (5.5 %) SIP changeovers (*P* = 0.01). Multivariate analysis identified two factors independently associated with a significant decrease in the risk of ≥20 % MAP drops, namely, SIP (OR, 0.47; 95 % CI, 0.22–0.98) and norepinephrine dose >0.5 μg/kg/min (OR, 0.39; 95 % CI, 0.19–0.81) (Table 3).

**Table 3:** Multivariate analysis to identify factors independently associated with mean arterial pressure (MAP) drops ≥20 % versus baseline

	OR [95 % CI]	*P* value
SIP changeover	0.47 [0.22–0.98]	0.04
Concentration of NE	0.95 [0.89–1.02]	0.19
Dose of NE >0.5 μg/kg/min	0.39 [0.19–0.81]	0.01
SOFA score at baseline	1.12 [0.99–1.26]	0.08
Age	1.00 [0.97–1.91]	0.85

*OR* odds ratio, *95* *% CI* 95 % confidence interval, *SIP* smart infusion pump, *NE* norepinephrine, *SOFA* Sequential Organ Failure Assessment

### Secondary outcomes

**Fig. 3:**
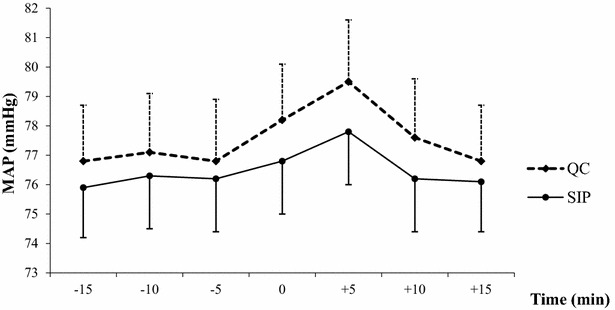
MAP variations before, during, and after norepinephrine changeovers. *MAP* mean arterial pressure, *QC* quick change, *SIP* smart infusion pump. Variations in mean values of MAP from 15 min before to 15 min after changeovers in both groups. T0 was just before changeover initiation

Overall, changeovers were associated with a significant MAP increase within the first 5 min, in both groups (QC group, from 78 ± 14 to 80 ± 14 mmHg, *P* = 0.03; SIP group, from 77 ± 13 to 78 ± 14 mmHg, *P* = 0.004) (Fig. 3). These increases were not significantly different between the two groups (*P* = 0.22). MAP increased by more than 20 % in a single QC patient and none of the SIP patients.

MAP drops ≥15 % occurred within 15 min after 40 (20.7 %) QC changeovers and 21 (9.6 %) SIP changeovers (*P* = 0.002). Similarly, MAP drops ≥10 % were significantly more common with QC than with SIP (34.2 vs. 24.3 %, *P* = 0.03). However, severe hypotension (MAP < 50 mmHg) occurred in similar proportions of patients in the two groups (4.7 % with QC and 4.1 % with SIP, *P* = 0.79).

HR changes ≥20 % occurred within 15 min after 15 (7.8 %) changeovers in the QC group (10 increases and 5 decreases) and 6 (2.8 %) changeovers in the SIP group (5 increases and 1 decrease) (*P* = 0.03). By multivariate analysis, the changeover method was not independently associated with HR variations (Table 4).

**Table 4:** Multivariate analysis to identify factors independently associated with heart rate (HR) changes ≥20 % in either direction versus baseline

	OR [95 % CI]	*P* value
SIP changeover	0.42 [0.12–1.40]	0.16
Concentration of NE	1.01 [0.92–1.11]	0.80
Dose of NE >0.5 μg/kg/min	0.37 [0.13–1.06]	0.06
SOFA score at baseline	1.03 [0.98–1.08]	0.26
Age	1.18 [0.97–1.42]	0.09

*OR* odds ratio, *95* *% CI* 95 % confidence interval, *SIP* smart infusion pump, *NE* norepinephrine, *SOFA* Sequential Organ Failure Assessment

## Discussion

We report the first prospective controlled trial designed to compare automated norepinephrine syringe changeovers using an SIP to manual changeovers using QC, in critically ill adults with shock. Our main finding is that SIP changeovers were associated with lower rates of hypotension than changeovers managed manually by the nurse using the QC technique.

Norepinephrine syringe changeovers are among the most important and stressful procedures required by the management of shock. There is a risk of decreased drug delivery during the changeovers [6, 8], with hypotension and clinical deterioration. Several methods have been developed to shorten the changeover period, thereby limiting the risks to the patient. However, few studies of these methods have been published. Manual changeover methods include the QC and the double infusion pump (DIP) technique. With both methods, the nearly empty syringe and the new full syringe, set at the same flow rate, are connected to the catecholamine line of the central venous catheter via a three-way stopcock, which is used to close the running infusion and open the new infusion at the same time. With QC, a brief interruption in the drug infusion may occur if the new line is not perfectly primed and/or the old syringe empties before the stopcock is turned to the new syringe and/or a delay occurs while turning the stopcock. Theoretically, none of these problems can occur with the DIP method, in which there is an overlap between the two syringes with a gradual decrease in the flow rate of the old syringe and a commensurate gradual increase in the flow rate of the new syringe. However, neither method is well standardized, a shortcoming that can result in flow rate variations, stress, human error, and hemodynamic instability. Previous studies found no significant differences in the risk of hemodynamic instability between QC and DIP [7, 9, 10]. Compared to DIP, QC is less time-consuming and therefore associated with a lower workload. We consequently chose QC as the comparator to SIP in our trial. SIPs were designed to drive syringe pumps and changeovers automatically according to specific algorithms to standardize drug delivery and decrease the nurse workload, thereby reducing the risk of error and improving the quality of care. However, their use remains controversial [11]. In some studies, the use of SIPs failed to improve drug safety [18, 19], due to the complexity of the SIP algorithm and settings. This complexity requires specific training of the nurses, which has been shown to decrease unwanted hemodynamic events after changeovers [7]. As indicated previously, all the nurses in our unit received in depth training and familiarization with both changeover methods. In a recent study, the use of an SIP for CVIP decreased the frequency of adverse hemodynamic events compared to QC [12]. However, the non-randomized before–after design of this study limits the interpretation of the results, as effects of time and other confounding factors cannot be excluded. Moreover, the patients received a variety of vasoactive drugs including norepinephrine, epinephrine, and dobutamine, whose different effects on α- and β-adrenergic receptors produce different changes in vascular tone, HR, and myocardial contractility [4] and, therefore, in blood flow and blood pressure. Thus, the variations in hemodynamic variables observed in this study [12] may be related to the changeover, drug effects, or both. In our trial, only norepinephrine was used. Norepinephrine chiefly induces vasoconstriction and is considered the vasopressor of choice for most critically ill patients with shock [1, 2, 20]. Last, in addition to the device or changeover technique, a major issue is standardization of the circuit to eliminate variations in dead volume, compliance, resistance, and flow rate [21]. In our study, the perfusion lines were set up in a standardized manner and in the same way in all study patients. In the light of these considerations, we are confident that the between-group differences in our study were ascribable only to the changeover technique.

The main finding from our trial is that using an SIP for norepinephrine syringe changeovers significantly decreased the frequency of MAP drops. This result is consistent with the above-mentioned before–after study, in which adverse hemodynamic events were also less common with SIP than with QC [12]. However, in contrast to previous studies, our trial did not include all adverse hemodynamic events (MAP drop, MAP elevation, and HR variation) in the primary endpoint [7, 12]. Instead, our primary endpoint was an MAP drop ≥20 %. A key goal of shock management is correction of the hypotension followed by prevention of further hypotensive episodes potentially responsible for decreased tissue perfusion. Current guidelines on shock recommend maintaining MAP at 65 mmHg or higher but do not indicate a maximum MAP value that should not be exceeded [2, 3]. Interestingly, as shown in Fig. 3, baseline MAP values in both groups were above the 65-mmHg minimal value indicated in our local shock management protocol. Moreover, MAP increased very slightly but significantly in both changeover groups. These findings are consistent with previous studies and reflect safety behaviors [7, 22] manifesting as a reluctance to decrease the norepinephrine flow rate even when the MAP target is reached in severely ill and unstable ICU patients. During changeovers, the mean MAP increase was also related to the brief increase in the norepinephrine flow rate due to the brief overlap between the nearly empty and full syringes. There is no evidence that moderate MAP increases have deleterious effects. A recent large randomized controlled trial in patients with septic shock found no significant differences in outcomes between the group with a high MAP target of 85–90 mmHg and the group with a standard MAP target of 65–70 mmHg [22]. Another finding from our study is that a norepinephrine dose >0.5 μg/kg/min was associated with a lower frequency of MAP drops. Thus, compared to lower flow rates, a high flow rate of norepinephrine protected against the small variations that may occur during changeovers. It is thus conceivable that maintaining a higher flow rate and using a more diluted norepinephrine solution may prevent MAP variations during changeovers in patients receiving low norepinephrine doses. However, higher flow rates may require a greater number of changeovers, and thereby increasing the risk of hemodynamic incidents. Last, norepinephrine has little effect on HR, which was therefore not included in our primary outcome. Our results consistently showed that the changeover method was not independently associated with HR variations.

Limitations of the trial include its single-center design, which may restrict the general applicability of the results. However, the devices and changeover methods used were very similar to those reported previously. Moreover, the patient characteristics reflected the overall population of patients with shock admitted to nonspecialized ICUs. Last, the prospective design strongly limits the risk of bias and allows the conclusion that using an SIP is causally related to a lower frequency of changeover-related hypotension. We are thus confident in the reliability and general applicability of our data. A second limitation of the trial is that, as indicated in the methods section, we stopped the randomization process when we found an imbalance between the two groups and we subsequently included all patients in the SIP group, to obtain similar numbers of changeovers in the two groups. We do not believe this fact significantly weakens our data, for the following reasons: (1) most patients were randomized, (2) consecutive patients were included, (3) all patients met predefined inclusion criteria, and (4) the multivariate analysis clearly demonstrated that the changeover method was independently associated with the occurrence of MAP variations. A third limitation is that we did not assess nurse workload, as our aim was to focus on potential benefits to patients. Although nurse workload is subjective, a previous non-randomized study showed a decrease in nurse’s workload with SIP use [12]. A fourth limitation of our study may lie in the method used to calculate the amount of norepinephrine in the syringe for each individual patient. Indeed, doses calculations may lead to errors. However, this method has been used for many years in our ICU. Thus, all the nurses have considerable experience with it, and we are confident that the risk of error was very low. Moreover, although smart pumps could calculate doses in µg/kg/min, they were not used in the QC group. We therefore used the calculation method in both groups to avoid concerns about possible differences in norepinephrine concentrations. Last, our trial was not designed to assess the potential impact of the changeover method on patient outcomes. A far larger number of patients would have been required to assess possible effects on mortality. However, maintenance of hemodynamic stability was associated with improved patient outcomes in previous work and is a key goal of critical care [23]. Thus, our trial can be viewed as an important first step in demonstrating that SIPs improve the quality of care. Our results support the conduct of a large trial designed to assess the impact of SIP norepinephrine changeovers on patient outcomes.

## Conclusion

This prospective controlled trial supports the use of SIPs for norepinephrine changeovers in critically ill adults with shock. Compared to QC, SIP changeovers are associated with improved hemodynamic stability.

## Abbreviations

ICU: intensive care unit
MAP: mean arterial pressure
NE: norepinephrine
QC: quick change
SIP: smart infusion pump

## References

[1] Bougle A, Harrois A, Duranteau J (2013). Resuscitative strategies in traumatic hemorrhagic shock. Ann Intensive Care..

[2] Dellinger RP, Levy MM, Rhodes A, Annane D, Gerlach H, Opal SM (2013). Surviving Sepsis Campaign: international guidelines for management of severe sepsis and septic shock, 2012. Intensive Care Med.

[3] Levy B, Bastien O, Benjelid K, Cariou A, Chouihed T, Combes A (2015). Experts’ recommendations for the management of adult patients with cardiogenic shock. Ann Intensive Care..

[4] Hollenberg SM (2011). Vasoactive drugs in circulatory shock. Am J Respir Crit Care Med.

[5] Monnet X, Lefrant JY, Teboul JL (2008). Field 6. Safety practices for haemodynamic procedures (administration of vasoactive drugs, vascular and cardiac catheterization). French-speaking Society of Intensive Care. French Society of Anesthesia and Resuscitation. Ann Fr Anesth Reanim.

[6] Trim JC, Roe J (2004). Practical considerations in the administration of intravenous vasoactive drugs in the critical care setting: the double pumping or piggyback technique-part one. Intensive Crit Care Nurs.

[7] Argaud L, Cour M, Martin O, Saint-Denis M, Ferry T, Goyatton A (2007). Changeovers of vasoactive drug infusion pumps: impact of a quality improvement program. Crit Care.

[8] Morrice A, Jackson E, Farnell S (2004). Practical considerations in the administration of intravenous vasoactive drugs in the critical care setting. Part II—how safe is our practice?. Intensive Crit Care Nurs.

[9] Arino M, Barrington JP, Morrison AL, Gillies D (2004). Management of the changeover of inotrope infusions in children. Intensive Crit Care Nurs.

[10] de Barbieri I, Frigo AC, Zampieron A (2009). Quick change versus double pump while changing the infusion of inotropes: an experimental study. Nurs Crit Care..

[11] Murdoch LJ, Cameron VL (2008). Smart infusion technology: a minimum safety standard for intensive care?. Br J Nurs..

[12] Cour M, Hernu R, Benet T, Robert JM, Regad D, Chabert B (2013). Benefits of smart pumps for automated changeovers of vasoactive drug infusion pumps: a quasi-experimental study. Br J Anaesth.

[13] Genay S, Decaudin B, Scoccia S, Barthelemy C, Debaene B, Lebuffe G (2015). An in vitro evaluation of infusion methods using a syringe pump to improve noradrenaline administration. Acta Anaesthesiol Scand.

[14] Ténière A, Omrani S, Odouard E, Szostek AS, Piriou V, Cabelguenne D (2015). Comparaison in vitro des dispositifs de perfusion : précision et fiabilité du débit, régulateur versus pince à roulette du perfuseur (In vitro comparison of infusion devices: accuracy and reliability of flow, infusion control device versus IV set). Anesth Reanim..

[15] Le Gall JR, Lemeshow S, Saulnier F (1993). A new Simplified Acute Physiology Score (SAPS II) based on a European/North American multicenter study. JAMA.

[16] Vincent JL, Moreno R, Takala J, Willatts S, De Mendonca A, Bruining H (1996). The SOFA (Sepsis-related Organ Failure Assessment) score to describe organ dysfunction/failure. On behalf of the Working Group on Sepsis-Related Problems of the European Society of Intensive Care Medicine. Intensive Care Med.

[17] Lakhal K, Ehrmann S, Runge I, Legras A, Dequin PF, Mercier E (2009). Tracking hypotension and dynamic changes in arterial blood pressure with brachial cuff measurements. Anesth Analg.

[18] Nuckols TK, Bower AG, Paddock SM, Hilborne LH, Wallace P, Rothschild JM (2008). Programmable infusion pumps in ICUs: an analysis of corresponding adverse drug events. J Gen Intern Med.

[19] Rothschild JM, Keohane CA, Cook EF, Orav EJ, Burdick E, Thompson S (2005). A controlled trial of smart infusion pumps to improve medication safety in critically ill patients. Crit Care Med.

[20] De Backer D, Biston P, Devriendt J, Madl C, Chochrad D, Aldecoa C (2010). Comparison of dopamine and norepinephrine in the treatment of shock. N Engl J Med.

[21] Peterfreund RA, Philip JH (2013). Critical parameters in drug delivery by intravenous infusion. Expert Opin Drug Deliv.

[22] Asfar P, Meziani F, Hamel JF, Grelon F, Megarbane B, Anguel N (2014). High versus low blood-pressure target in patients with septic shock. N Engl J Med.

[23] Beck V, Chateau D, Bryson GL, Pisipati A, Zanotti S, Parrillo JE (2014). Timing of vasopressor initiation and mortality in septic shock: a cohort study. Crit Care.

